# HEALTHY AGING DURING THE PANDEMIC AND BEYOND: LESSONS FROM FIVE YEARS OF THE NATIONAL POLL ON HEALTHY AGING AND AARP

**DOI:** 10.1093/geroni/igac059.902

**Published:** 2022-12-20

**Authors:** Erica Solway, Teresa Keenan, Brian Lindberg

## Abstract

The past five years have brought enormous changes in the lives of older adults and their families. During this time the University of Michigan National Poll on Healthy Aging (NPHA), co-sponsored by AARP and Michigan Medicine, has gathered the experiences and perspectives from nationally representative samples of US adults age 50-80 on a range of topics related to healthy aging. These polls have been used to elevate the voices of the older adults in the development of policies and practices to improve the health of people as they age. During the past two years, poll reports have captured how changes in lifestyle and behaviors during the COVID-19 pandemic have impacted the health and well-being of older adults. Members of the NPHA team from the University of Michigan and AARP will begin with an overview of the poll including its goals and methods. The session will then highlight key findings from the first five years of the NPHA that have important implications during and beyond the COVID-19 pandemic, with special attention to poll findings related to joys and stresses and aging in place. The presenters will also discuss how to access publicly available NPHA data. The session will wrap up with a discussion of how the perspectives and experiences of older adults gathered through the NPHA and AARP polling can be used to inform policy and advocacy to support healthy aging.

